# The Interaction of Apical Periodontitis, Cigarette Smoke, and Alcohol Consumption on Liver Antioxidant Status in Rats

**DOI:** 10.3390/ijms252212011

**Published:** 2024-11-08

**Authors:** Danilo Cassiano Ferraz, Camilla Christian Gomes Moura, Nara Sarmento Macêdo Signorelli, Rodrigo César Rosa, Sanívia Aparecida de Lima Pereira, Ana Luiza Silva Borges, Vinícius Prado Bittar, Rener Mateus Francisco Duarte, Renata Roland Teixeira, Martinna Bertolini, Foued Salmen Espindola

**Affiliations:** 1 Department of Endodontics, School of Dentistry, Federal University of Uberlândia, Uberlândia 38405-266, MG, Brazil; daniloferraz@ufu.br (D.C.F.);; 2 Institute of Biological and Natural Sciences, Federal University of Triângulo Mineiro, Uberaba 38025-180, MG, Brazil; 3 Laboratory of Biopathology and Molecular Biology, University of Uberaba, Uberaba 38055-500, MG, Brazil; 4 Institute of Biotechnology, Federal University of Uberlândia, Uberlândia 38405-319, MG, Brazilfoued@ufu.br (F.S.E.); 5 Department of Periodontics and Preventive Dentistry, University of Pittsburgh, Pittsburgh, PA 15261, USA

**Keywords:** animal model, antioxidants, alcoholism, apical periodontitis, cigarette smoking, chemical and drug-induced liver injury, oxidative stress

## Abstract

This study aimed to investigate the impact of alcohol (A), secondhand cigarette smoking (ShS), and their combined effect on liver antioxidant activity and hepatic damage in rats with induced apical periodontitis (AP). Thirty-five female Wistar rats were randomly allocated into five groups (n = 7): (1) control (rats without ShS, alcoholic diet, or AP), (2) control-AP (induced AP only), (3) ShS-AP (ShS exposure and induced AP), (4) A-AP (alcoholic diet and induced AP), and (5) A+ShS-AP (alcoholic diet, ShS exposure, and induced AP). Alcohol was administered through semi-voluntary intake, while ShS exposure involved the daily inhalation of cigarette smoke. The experimental period lasted 8 weeks, with AP induction occurring in the 4th week following molar pulp exposure. Liver samples were collected post-euthanasia for histomorphometric and antioxidant marker analyses. All AP-induced groups exhibited increased liver sinusoidal dilation compared to the control group (*p* < 0.05). AP significantly reduced total antioxidant capacity (FRAP) across all groups (*p* < 0.05). In AP-induced groups, FRAP levels were further decreased in ShS-AP and A+ShS-AP compared to control-AP (*p* < 0.05). AP also led to a decrease in the glutathione defense system (*p* < 0.05). Rats with alcohol exposure (A-AP and A+ShS-AP) showed reduced glutathione peroxidase activity (*p* < 0.05). Glutathione reductase activity was comparable in the control and control-AP groups (*p* > 0.05), but significantly decreased in the alcohol and ShS-exposed groups (*p* < 0.05). Apical periodontitis can relate to morphological changes in the liver’s sinusoidal spaces and impairment of liver’s antioxidant capacity of rats, particularly when combined with chronic alcohol consumption and exposure to cigarette smoke.

## 1. Introduction

Excessive alcohol consumption and tobacco smoking are major contributors to preventable deaths worldwide, exerting detrimental effects on both quality of life and the global economy [1,2]. Despite the social acceptance of alcohol consumption, chronic use adversely affects organs such as the brain, muscles, bones, and liver, compromising the immune system and increasing susceptibility to severe infections [3,4,5,6]. Similarly, exposure to environmental tobacco smoke, also known as secondhand smoke (ShS), has been implicated in the development of organ damage. This occurs through mechanisms involving inflammatory responses and disruptions in metabolic pathways, particularly affecting the liver [7,8]. Such disruptions can contribute to the onset of conditions including non-alcoholic fatty liver disease, liver fibrosis, and an increased risk of hepatocellular carcinoma [9,10].

Both experimental and epidemiological studies [7,11] highlight a synergistic relationship between alcohol abuse and tobacco smoking in the onset and progression of liver disease. However, the precise mechanisms underlying these interactions and the resulting liver damage remain incompletely understood [11]. The overproduction of free radicals, particularly reactive oxygen species (ROS), is considered a key factor in the damage induced by both alcohol abuse and tobacco smoking through oxidative stress (OS) [12]. An impaired antioxidant defense system contributes to OS, which in turn modulates immune responses, influences inflammatory processes, and hampers tissue repair [13]. Thus, preserving a balance between oxidants and antioxidants by strengthening the body’s innate antioxidative defense mechanisms is crucial for overall health. Mammals developed a complex enzymatic and non-enzymatic antioxidant system to relieve OS [14]. The reduced glutathione (GSH) is the most abundant antioxidant in human cells, which serves as a free radical scavenger and detoxifier [15]. Additionally, endogenous antioxidant enzymes, such as superoxide dismutase (SOD), glutathione peroxidase (GPx), glutathione reductase (GR), and glucose-6-phosphate dehydrogenase (G6PD), play a key role in modulating liver disease [16].

Chronic consumption of alcohol and tobacco not only inflicts OS-related damage to the liver, but also compromises the immune system [6,17], making individuals more susceptible to infections like apical periodontitis (AP). AP is an inflammatory condition triggered by bacterial infections in the dental pulp, leading to periapical tissue destruction [18]. Existing studies in both animals [19,20,21,22,23] and humans [24,25,26,27] indicate elevated levels of local and systemic ROS in response to AP [21,22,28], which disturb the redox balance in the body [21,22].

During phagocytosis, reactive molecules are produced and converted into other reactive species that act as both signaling molecules, activating protective cellular pathways, and as agents that destroy pathogens [29]. After phagocytosis, protective mechanisms, commonly referred to as antioxidants, work to restore the balance by reversing any oxidative damage to biological targets, while excess reactive molecules are neutralized by specific enzymes [29,30]. This controlled oxidative response, known as “oxidative eu-stress”, is essential for maintaining immune defense. However, if the system is overwhelmed by aggressive pathogens or impaired antioxidant capacity, the oxidative process can become uncontrolled, resulting in “oxidative di-stress” [30]. This imbalance leads to damage to surrounding tissues, including the periapical region, contributing to the progression of AP and posing broader health risks [9,28].

Animal studies have demonstrated that AP may damage organs, altering OS parameters in the heart [19,31], kidneys [19], and liver [19,32,33]. As the liver plays a critical role in detoxifying harmful substances and managing OS, the combined impact of alcohol, tobacco, and chronic infections such as AP can exacerbate hepatic damage [3,7,22]. This synergy between unhealthy habits and chronic infections may suggest the need for a deeper understanding of these interactions to develop more effective prevention strategies. It underscores the interconnected nature of OS and the importance of addressing all contributing factors, including oral health, in patients with chronic alcohol or tobacco use.

To date, there is a lack of studies investigating the effects of AP, cigarette smoke, and chronic alcohol consumption on hepatic tissue impairment. Therefore, this study aimed to assess the effects of alcohol, ShS, or their combination on liver antioxidant activity and morphological changes in rats with induced AP.

## 2. Results

### 2.1. Clinical Observations

**Figure 1:**
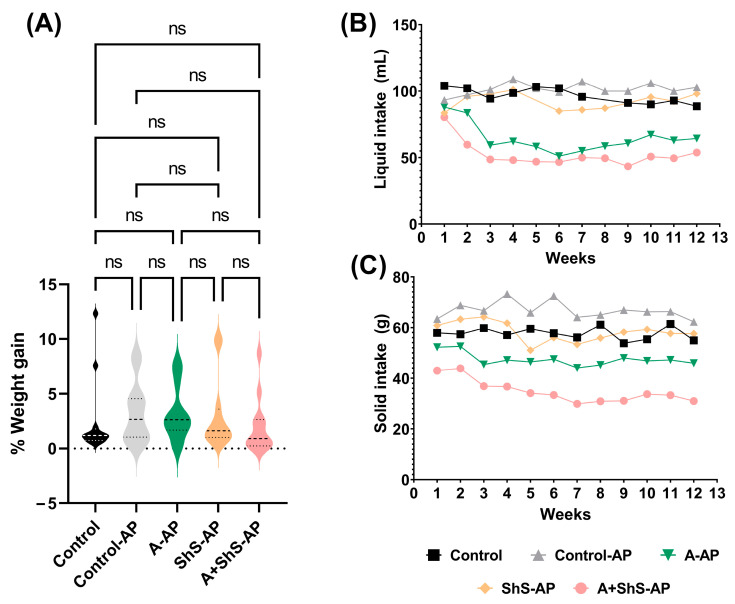
Clinical observations during the experimental period. (**A**) Representative percentage of weight gain in rats throughout the study (*p* > 0.05, one-way repeated-measures ANOVA followed by Tukey’s post hoc test). (**B**) Liquid and (**C**) solid intake by rats over time. Intake data are presented as the mean weekly consumption values for the following groups: control (■), control-AP (▲), A-AP (▼), ShS-AP (♦), and A+ShS-AP (●).

**Figure 2:**
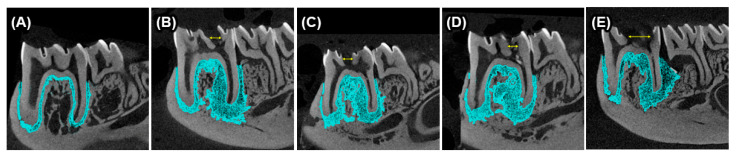
Axial micro-CT reconstructions showing (**A**) no AP development in the control group and confirmed AP development in the (**B**) A-AP, (**C**) ShS-AP, (**D**) A-AP and (**E**) A+ShS-AP groups. Yellow arrows indicate the coronal opening used to induce apical lesions. Blue hatched areas represent the extent of the apical lesion (**B**–**D**) compared to the absence of lesions and normal periodontal ligaments in the control group (**A**).

The animals weighed 154.9 g (±12.9 g) at the start of the experiment, with no adverse events or losses. Weight gain was unaffected by the experimental design throughout the study period (Figure 1A). Weekly solid and liquid intake are provided in Figure 1B,C. The AP lesions were confirmed through micro-CT reconstructions, as shown in Figure 2.

### 2.2. Liver Fibrosis Analysis

**Figure 3:**
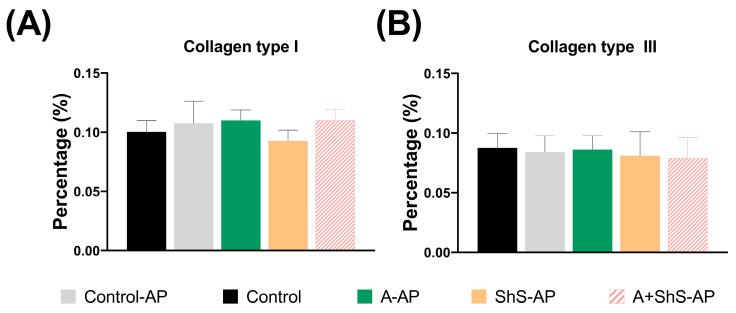
Percentage of collagen type I (**A**) and III (**B**) in the livers of rats. Data are presented as mean ± SD.

No significant fibrosis was induced by AP or deleterious habits. There were no significant differences in collagen type I and III deposition among all groups (*p* > 0.05) (Figure 3A,B).

### 2.3. AP Is Related to Hepatic Sinusoidal Dilation

**Figure 4:**
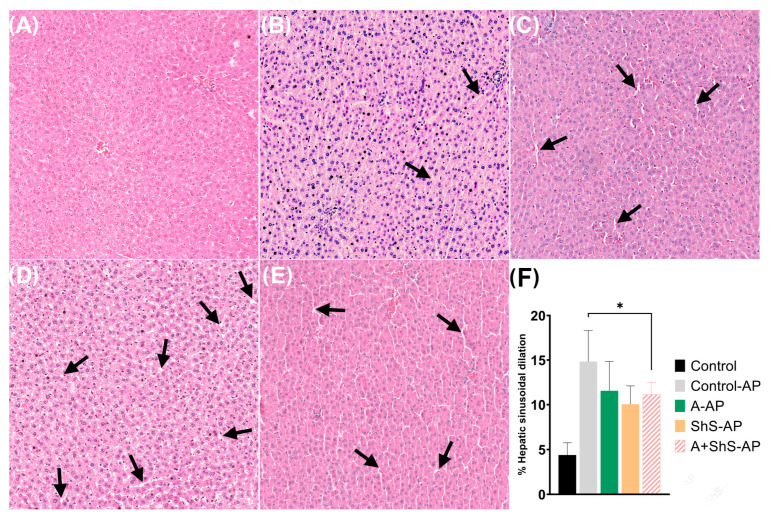
Photomicrographs of liver sections evaluating hepatic sinusoidal dilation from the (**A**) control, (**B**) control-AP, (**C**) A-AP, (**D**) ShS-AP, and (**E**) A+ShS-AP groups (20× magnification). The hepatic sinusoidal dilation values are expressed in percentages (**F**). Note the sinusoidal dilation by black arrows. * *p* < 0.05 vs. control group.

The results of the liver sinusoidal analysis are summarized in Figure 4. All experimental groups exhibited significantly higher percentuals of hepatic sinusoidal dilation compared to the control group (*p* < 0.001). Among the AP-induced groups, there were no significant differences in sinusoidal dilation percentage (*p* > 0.05).

### 2.4. AP Alters the Antioxidant Capacity of Liver

**Figure 5:**
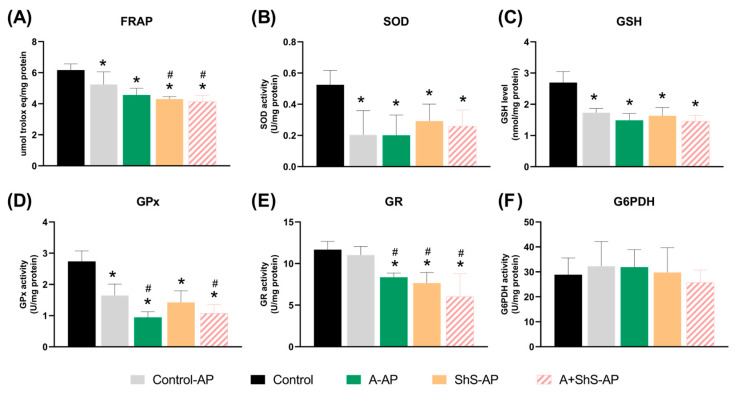
Assessment of (**A**) overall antioxidant capacity, (**B**) superoxide dismutase, (**C**) reduced glutathione, (**D**) glutathione peroxidase, (**E**) glutathione reductase, and (**F**) glucose 6-phosphate dehydrogenase. The values are presented as mean ± standard deviation. * *p* < 0.05 vs. control group, # *p* < 0.05 vs. control-AP.

The antioxidant capacity in the rats’ livers is shown in Figure 5. The total antioxidant capacity (FRAP) decreased significantly (*p* < 0.05) in all AP-induced groups compared to the control group. Within the AP-induced groups, FRAP levels were significantly lower in the ShS-AP and A+ShS-AP groups compared to the control-AP group (Figure 5A). SOD levels decreased significantly in all AP-induced groups compared to the control (*p* < 0.05, Figure 5B).

The GSH concentration and GPx enzyme activity significantly decreased (*p* < 0.05) in all AP-induced groups compared to the control group (Figure 5C). Among animals exposed to chronic alcohol consumption (A-AP and A+ShS-AP), GPx enzyme activity also decreased significantly (*p* < 0.05) compared to the control-AP group (Figure 5D). The activity of the GR enzyme (Figure 5E) decreased in all animals exposed to harmful habits (ShS-AP, A-AP, and A+ShS-AP) compared to both control groups (control and control-AP). The G6PD activity (Figure 5F) was similar in all tested groups (*p* > 0.05).

## 3. Discussion

The crosstalk between AP and liver damage has gained attention due to emerging evidence from clinical studies linking AP prevalence to liver impairment [34,35,36] and altered liver markers in animal models [19,33,37]. The present study provides new evidence that AP can be related to sinusoidal dilation and a reduction in antioxidant capacity in rat liver tissue, as evidenced by decreased levels of FRAP, SOD, GSH, GR, and GPx. Furthermore, chronic alcohol consumption and exposure to ShS intensified the reductions in FRAP and GPx levels.

Animal models, particularly rodents, are crucial for investigating the systemic effects of AP, including its role in OS [19]. While clinical studies have examined the local and systemic impacts of AP on oxidative imbalance in human biological fluids such as blood, saliva, and crevicular fluid [18,21], animal models allow for controlled experimentation on the effects of AP on target organs like the liver. The rat has been the main animal model for mimicking human AP [20,23,38], facilitating detailed assessments of systemic outcomes [19].

This study revealed a disruption in antioxidant balance in hepatic tissue, which was related to a potential risk of AP, alcohol consumption, and ShS to the impairment of antioxidant balance. However, no significant fibrosis was developed in any group, since our study model did not use highly hepatotoxic substances. Previous studies linking liver fibrosis with AP have utilized potent hepatotoxic agents, such as carbon tetrachloride, over extended periods (e.g., 60 days) to induce fibrosis [38,39]. However, the primary objective of this study was not to replicate those fibrosis-inducing models, since prolonged exposure to highly hepatotoxic substances would not allow for the evaluation of AP’s effect on liver fibrosis. Additionally, our study was conducted over a shorter experimental period and used a model that induced AP in only one tooth. Studies have shown that inducing AP in more than one tooth increases systemic inflammatory load [40], which may have contributed to the lack of histopathological changes consistent with fibrosis in our model. Although cigarette smoke and chronic alcohol consumption are recognized contributors to hepatic fibrosis [10,41], our study used a lower exposure of 4 cigarettes/day for ShS compared to 10 cigarettes/day in previous AP models [17,42]. Additionally, we implemented semi-voluntary alcohol consumption, which reduced solid and liquid intake in the animals. A similar reduction in appetite and fluid intake was reported in another alcohol consumption model [43,44]. These factors likely contributed to the absence of fibrosis in our study.

In addition to fibrosis analysis, another commonly evaluated parameter for hepatic alterations is sinusoidal dilation [45]. Regarding the morphological changes, all AP groups in this study exhibited greater sinusoidal dilation compared to the control group. Most of the blood flow to the central vein directly depends on the sinusoids, where the majority of physiological exchanges between the blood and liver cells occur [45]. When dilated, sinusoids are a significant risk factor for impaired portal venous inflow, which may contribute to systemic inflammatory response syndrome [46].

In animal studies, AP lesions combined with chronic alcohol consumption and nicotine injections alter metabolic markers, impair liver function, and increase systemic proinflammatory mediators [47]. Although our study did not use nicotine injections, nicotine is a primary component of cigarette smoke and is reported to cause peripheral vasoconstriction, limiting oxygen supply to tissues [47]. While nicotine injection models are supported in the literature [47,48], they do not fully replicate ShS exposure, as they do not provide the smoke directly to the animal and cannot be directly correlated with an ShS model. Additionally, smoking has been linked to sarcopenia in individuals over 60 years of age, with risk severity correlating with both the duration and cumulative dose of smoking [49]. This condition is closely associated with increased ROS levels, highlighting OS as a contributing factor [50]. Sarcopenia is a frequent complication of chronic liver disease, serving as an independent predictor of mortality in patients with cirrhosis [50,51]. Furthermore, sarcopenia is associated with a high prevalence of periodontal disease—a condition with pathogenic mechanisms similar to AP [52]—which may support the translational relevance of our findings from this animal model to human disease contexts.

The systemic production of ROS by AP is well established [21,22,28], although less emphasis has been placed on the impact of AP on antioxidant balance. In this study, the reduced levels of FRAP, SOD, GSH, and GPx in the livers of the AP groups suggest that the weakened antioxidant defenses may impair the liver’s ability to neutralize ROS. The decline in FRAP, SOD, and GPx reflects weakened enzymatic defenses against superoxide radicals and hydrogen peroxide [53,54]. The drop in GSH indicates depleted non-enzymatic antioxidant reserves, further worsening oxidative damage [55]. The greater reduction in GPx in AP groups exposed to alcohol points to a combined effect of AP and alcohol-induced OS [46]. Reduced GR levels have been confirmed in animal models exposed to alcohol and cigarette smoke, further promoting OS [56]. Our results showed a greater reduction in FRAP levels of both groups exposed to ShS. Nicotine injections have been shown to significantly reduce FRAP levels in liver tissue compared to non-injected animals [57]. Although nicotine is a known inducer of OS in injection models [58], our study demonstrated that the ShS model also reduced FRAP levels.

The present study demonstrated a reduction in biomarker levels in the AP model, consistent with the findings of Xiao et al. (2023) [33] and Barcelos et al. (2020) [19]. Although these studies did not assess the same biomarkers, Xiao et al. [33] reported an increased OS-index based on the total oxidant-to-antioxidant ratio in the liver, along with reduced total antioxidant capacity in the serum of rats with AP. Additionally, we observed a decrease in SOD and GPx activities, while Barcelos et al. [19] found decreased catalase activity in the livers of rats with AP. The impact of AP on antioxidant balance extends beyond the liver, affecting other systemic targets such as the parotid gland [59], heart, and pancreas [19,31]. When exposed to factors like ShS and alcohol, our study revealed greater reductions in FRAP, GPx, and GR levels, suggesting a synergistic effect between AP, ShS, and chronic alcohol consumption.

Some limitations of this study should be addressed, such as the fact that the semi-voluntary alcohol consumption model may lead to dehydration when alcohol is the only source of fluid intake. Moreover, the study did not evaluate specific markers of fibrosis, a condition influenced by OS, such as TGF-β1 and the activation of Kupffer cells, which release inflammatory mediators like IL-1β and TNF-α [38]. Future research should prioritize identifying specific OS markers to detect oxidative distress more accurately in liver tissue, in addition to assessing antioxidant capacity. Moreover, molecular analysis of hepatic tissue, including gene expression and immunostaining, would provide further insights into the underlying mechanisms of liver damage, along with three-dimensional localization within the tissue. We emphasized the importance of employing established techniques to verify our results in the revised manuscript.

Additionally, the primary aim of this study was to evaluate the effects of AP, alcohol consumption, and ShS on liver tissue in rats. While the impact of chronic alcohol consumption and cigarette smoke exposure is well-documented, further investigation is needed to understand the combined effects of these factors on other target organs, particularly given the increasing evidence linking AP to systemic health issues. Another limitation is the use of a female rodent model. Although animal models are commonly used for AP studies and provide effective control over AP development, they may not fully mimic human pathophysiology. Moreover, not accounting for sex differences in response to the factors studied may limit the extrapolation of our results. This study provides a foundation for future research that includes both sexes, highlighting the importance of sex-specific studies in developing targeted therapeutic strategies. Such research will advance our understanding of how liver responses vary under complex environmental and pathological stressors. Future studies incorporating both sexes will be essential to confirm whether these patterns remain consistent across biological variations. Translating these results to clinical practice will require careful interpretation and validation through well-designed clinical trials, as well as new models to assess the impact of these factors on human diseases such as sarcopenia.

## 4. Materials and Methods

### 4.1. Animals

Approval for the experimental protocol was obtained from the Ethics Committee for Animal Use at UNIUBE, Uberaba, Brazil (Protocol No. 001/2021), following the Preferred Reporting Items for Animal Studies in Endodontology (PRIASE) guidelines [60]. Thirty-five female Wistar rats, 60 days old and weighing approximately 150 g each, were utilized. Sample size calculation considered a minimum treatment difference of 1.53 and a standard deviation of 0.61 [61]. This calculation determined seven rats per group, with a test power of 95% and a significance level of 5%.

The rats were housed in polypropylene cages in a temperature-controlled environment (22–25 °C), subjected to 12 h light and dark cycles with approximately 55% air humidity, and provided with ad libitum access to food and water. Daily clinical monitoring was performed to detect signs of animal distress. Weekly weight measurements were taken using a semi-analytical scale. Daily solid and liquid intake were recorded. The animals were cared for by facility staff under veterinary supervision. Euthanasia was performed via intraperitoneal administration of a lethal dose of thiopental at the end of the experiment.

### 4.2. Alcohol Consumption and Smoke Inhalation

Animals were randomly assigned to one of five experimental groups (n = 7 per group): (1) control (rats not exposed to ShS or an alcoholic diet and without AP); (2) induced AP (control-AP); (3) exposed to ShS and induced AP (ShS-AP); (4) exposed to an alcoholic diet and induced AP (A-AP); and (5) exposed to an alcoholic diet plus ShS and induced AP (A+ShS-AP).

**Figure 6:**
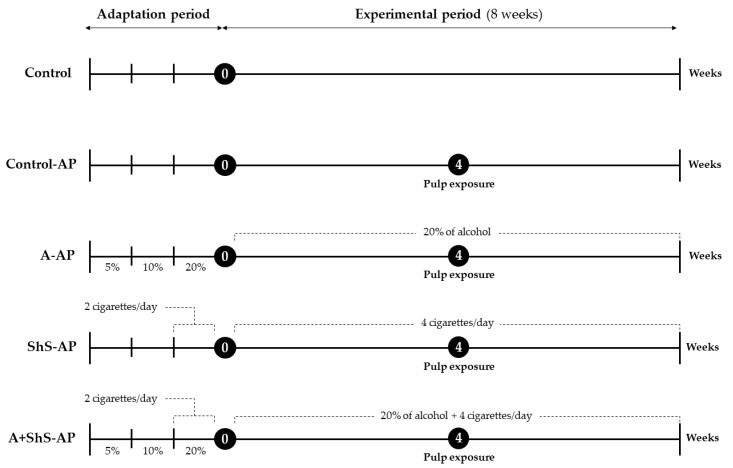
Experimental design flowchart. The study included a 3-week adaptation period with gradually increased alcohol consumption and ShS exposure, followed by an 8-week experimental period. AP was induced through pulp exposure in the 4th week of the experimental period. The animals were euthanized 4 weeks after AP induction.

The experimental model of alcohol exposure adopted was semi-voluntary intake [44,62,63]. Therefore, the animals were subjected to a chronic alcohol consumption induction protocol (CIP). In order to induce chronic ethanol consumption, the animals received 5% ethyl alcohol diluted in water in the first week. In the following week, they received 10% ethyl alcohol, and in the third and fourth weeks, 20% ethyl alcohol. After CIP, the animals continued to receive a liquid diet with 20% ethyl alcohol for another eight weeks until euthanasia (Figure 6). The alcoholic solution was the only source of liquid intake throughout the entire experimental procedure.

Cigarette smoke exposure, aiming to simulate conditions of ShS, was conducted following the methodology outlined by Santiago et al. [64]. A transparent acrylic box containing four cylindrical inhalation chambers was employed (7.0 cm in diameter and 23.0 cm in length, with a total volume of 885 cm^3^). One end of the chamber was accessible for animal handling, while the opposite funnel-shaped end was linked to a peristaltic pump. This pump drew in the smoke and channelled it through a distributor into the animal chambers. During exposure, four animals were positioned with their noses directed toward the cylindrical end, securely held in place by a restrainer. The source of the smoke was Marlboro cigarettes (Philip Morris, Richmond, VA, USA). Each unit, as indicated on the product label, contained 0.8 mg of nicotine, 10 mg of tar, and 10 mg of carbon monoxide. To ensure consistent exposure to tobacco smoke, the carbon monoxide levels in each chamber of the equipment were measured daily using a portable carbon monoxide meter (Instrutemp ITMCO-1500, São Paulo, SP, Brazil). The average carbon monoxide levels were consistent across all measurements, with a mean of 338.79 ± 1.16 ppm.

Animals were introduced to the cylindrical chambers without cigarette smoke for one week to acclimate. In the second week, smoke inhalation began by burning one cigarette twice daily (morning and afternoon, with a 6 h interval). At the third week, exposure increased to four cigarettes per day (two in the morning and two in the afternoon, with a 6 h interval). At this point, the baseline was determined, and the exposure regimen persisted until euthanasia. The animals not submitted to smoke inhalation were exposed to smoke-free chambers in order to be submitted to the same type of stress. Details are summarized in Figure 6.

### 4.3. Apical Periodontitis Induction

AP was induced in the fourth week by occlusal pulpal exposure of the lower right first molars under anesthesia (intramuscular ketamine/xylazine at doses of 70 and 6 mg/kg, respectively). Pulpal exposure was performed using a round bur with a 0.1 mm diameter (Dentsply Sirona, Ballaigues, Switzerland) operating at high speed with continuous irrigation. The pulp chambers were left open to the oral environment for four weeks to allow for AP development (Figure S1). AP development was confirmed after euthanasia by a micro-computed tomography device (micro-CT, Bruker, Kontich, Belgium), with an Al 1 mm filter operating at 80 kV voltage, uA current, and a resolution of 9 µm. The scan datasets were reconstructed using NRecon software (v.1.6.10.4, Bruker) with a ring artifact correction set to 4. Axial views of the datasets were obtained using DataViewer software (v.1.5.2.4, Bruker) for observation.

### 4.4. Sample Collection and Histological Processing

The left liver lobes were collected and immersed in a buffered solution containing 4% paraformaldehyde at a neutral pH for 48 h for histological analysis. Simultaneously, the right lobes were frozen at −80 °C for subsequent biochemical analysis. Fixed specimens underwent conventional histological processing and paraffin embedding. Slices with 5 µm thickness were obtained using a microtome (Leica Biosystems RM2245, Leica, Nussloch, Germany). Hematoxylin and eosin (H&E) staining was conducted on two sequential sections, while Picrosirius Red (PS) staining was performed on two additional sections. A 50 µm interval was maintained before obtaining the next sections, resulting in a semi-serial approach. Ten sections of each staining type were obtained per animal.

### 4.5. Histomorphometric Analyses

PS-stained sections were captured using a polarized light microscope (Nikon Eclipse Ti-S, Tokyo, Japan) at 20× magnification. Images were assessed for total collagen content in four distinct fields per section, with a total of five sections per animal evaluated, resulting in 140 analyses per group. Total collagen fibers were quantified using ImageJ software, and the percentages of type I and type III collagen (reddish-yellow and whitish-green birefringence, respectively) were determined. The ImageJ software (ImageJ 1.51k, Wayne Rasband, National Institute of Health, Bethesda, MD, USA) was calibrated using the Split Channel and Threshold tools, ensuring that all images were adjusted to capture the same color range. The hepatic capsule and major portal tracts were excluded from the analysis as they did not represent collagen deposition associated with hepatic damage [65].

H&E-stained sections were captured using a digital scanner coupled with a microscopic camera (ScanScopeAT; Leica Biosystems Imaging, Nussloch, Germany) with a 20× objective lens. The same pattern as described for PS-stained sections was followed for H&E-stained sections. The stained areas within the sinusoids were removed using Photoshop v. 24.x (Adobe Systems, San Jose, CA, USA), and images were converted into binary format using ImageJ to calculate sinusoidal dilation. All analyses were manually performed using ImageJ software. In both histomorphometric analyses, the percentage of each collagen type and the percentage of area occupied by the sinusoids obtained from each image were tabulated, generating an average per section and per animal.

### 4.6. Antioxidant Status Assays

Liver tissue was homogenized in ice-cold phosphate buffer (pH 7.4) and subsequently centrifuged at 800× *g* for 10 min at 4 °C. Protein levels were quantified using the Bradford method [66]. Total antioxidant capacity was assessed using the ferric reducing antioxidant power (FRAP) technique [67]. To measure the amount of reduced glutathione (GSH), the proteins of the samples were first extracted by adding metaphosphoric acid (1:1) and then centrifuging the mixture at 7000× *g* for 10 min. The resulting supernatants were then mixed with o-phthaldialdehyde (1 mg/mL), and the fluorescence was measured at an excitation wavelength of 350 nm and an emission wavelength of 420 nm in 96-well microplates (PerkinElmer LS 55, Waltham, MA, USA); GSH content was determined according to an analytical curve constructed with GSH [68]. Glutathione peroxidase (GPx) activity was determined according to the nicotinamide adenine dinucleotide phosphate (NADPH) decay for 10 min in 96-well microplates, then read at 340 nm [69]. Glutathione reductase (GR) activity was measured according to the consumption of NADPH by oxidized glutathione; NADPH decay was monitored for 10 min, with the absorption being measured at 340 nm [70]. Superoxide dismutase (SOD) activity (U/mg protein) was measured by inhibiting the autoxidation of pyrogallol [71].

### 4.7. Statistical Analysis

Normality was assessed using the Shapiro–Wilk test. One-way ANOVA followed by Dunnett’s post hoc test for multiple comparisons against the control group and Tukey’s test for multiple comparisons between groups were employed. Data were analyzed using GraphPad Prism 9.5.1 (GraphPad Software, Boston, MA, USA) at a 5% significance level.

## 5. Conclusions

This study assessed the effects of alcohol, ShS, and their combination on liver antioxidant activity and hepatic damage in rats with induced AP. Our findings suggest a significant association between apical periodontitis, chronic alcohol consumption, and secondhand smoke exposure with reductions in liver antioxidant capacity and morphological changes. The combination of AP with chronic alcohol consumption and ShS exposure further reduced antioxidant levels. These results underscore a molecular and pathophysiological association between oral health and systemic disease, highlighting a potential increased risk of liver dysfunction when AP is combined with common lifestyle factors.

## Data Availability

The raw data from this study are included in the article/Supplementary Materials. Further inquiries can be directed to the corresponding authors.

## Supplementary Materials

The following supporting information can be downloaded at: https://www.mdpi.com/article/10.3390/ijms252212011/s1.
