# Supplementary material for: The Interaction of Apical Periodontitis, Cigarette Smoke, and Alcohol Consumption on Liver Antioxidant Status in Rats
Source: Int J Mol Sci. 2024 Nov 8;25(22):12011. doi: 10.3390/ijms252212011 (PMC11593682; doi:10.3390/ijms252212011)
Supplement: Supplementary file 1 [file ijms-25-12011-s001.zip › Figure S1.docx]

**
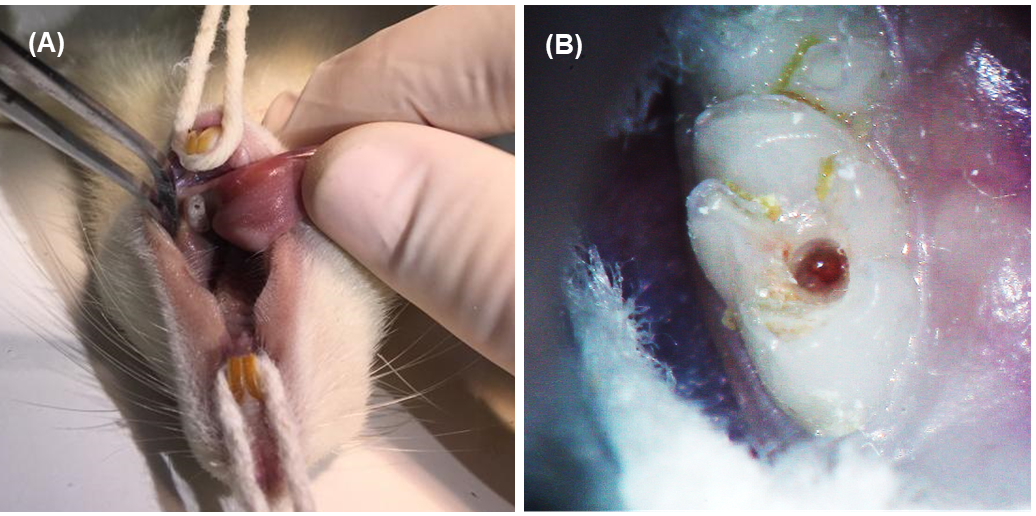
**

**Figure S1.** Induction of apical periodontitis through (**A**) animal immobilization after anesthesia and (**B**) pulp chamber exposure (20× magnification).
